# 

**Published:** 1903-04-04

**Authors:** 


No. 862 Vol. XXXIV. [Registered as a Newspaper.] SATURDAY, APRIL 4TH, 1903. [Subscription Terms, see p. 4.] PRICE THREEPENCE.

